# Correlations between predicted protein disorder and post-translational modifications in plants

**DOI:** 10.1093/bioinformatics/btt762

**Published:** 2014-01-07

**Authors:** Atsushi Kurotani, Alexander A. Tokmakov, Yutaka Kuroda, Yasuo Fukami, Kazuo Shinozaki, Tetsuya Sakurai

**Affiliations:** ^1^RIKEN Center for Sustainable Resource Science 1-7-22 Suehiro-cho, Tsurumi-ku, Yokohama 230-0045, Japan, ^2^Department of Biotechnology and Life Science, Faculty of Technology, Tokyo University of Agriculture and Technology, 2-24-16 Naka-cho, Koganei, Tokyo 184-8588, Japan and ^3^Research Center for Environmental Genomics, Kobe University, 1-1 Rokko dai, Nada, Kobe 657-8501, Japan

## Abstract

**Motivation:** Protein structural research in plants lags behind that in animal and bacterial species. This lag concerns both the structural analysis of individual proteins and the proteome-wide characterization of structure-related properties. Until now, no systematic study concerning the relationships between protein disorder and multiple post-translational modifications (PTMs) in plants has been presented.

**Results:** In this work, we calculated the global degree of intrinsic disorder in the complete proteomes of eight typical monocotyledonous and dicotyledonous plant species. We further predicted multiple sites for phosphorylation, glycosylation, acetylation and methylation and examined the correlations of protein disorder with the presence of the predicted PTM sites. It was found that phosphorylation, acetylation and O-glycosylation displayed a clear preference for occurrence in disordered regions of plant proteins. In contrast, methylation tended to avoid disordered sequence, whereas N-glycosylation did not show a universal structural preference in monocotyledonous and dicotyledonous plants. In addition, the analysis performed revealed significant differences between the integral characteristics of monocot and dicot proteomes. They included elevated disorder degree, increased rate of O-glycosylation and R-methylation, decreased rate of N-glycosylation, K-acetylation and K-methylation in monocotyledonous plant species, as compared with dicotyledonous species. Altogether, our study provides the most compelling evidence so far for the connection between protein disorder and multiple PTMs in plants.

**Contact:** tokmak@phoenix.kobe-u.ac.jp or tetsuya.sakurai@riken.jp

**Supplementary information:** Supplementary data are available at *Bioinformatics* online.

## 1 INTRODUCTION

Proteins with long disordered regions, often referred to as intrinsically disordered regions (IDRs), have been extensively investigated over the past several years. They represent a broad class of proteins found more abundantly in eukaryotes than in bacteria or archaea (Dunker *et al.*, 2001). It was estimated that more than one-third of eukaryotic proteins contain IDRs of >30 residues in length (Ward *et al.*, 2004a). Other bioinformatics studies indicated that more than half of eukaryotic proteins possess IDRs and ∼25–30% of the proteins are mostly disordered (Oldfield *et al.*, 2005a and b; Uversky, 2011). The proteins with IDRs are also widespread in plant proteomes with the overall proteome disorder content in different plant species being typical for other eukaryotes (Dunker *et al.*, 2001; Fukuchi *et al.*, 2011; Xue *et al.*, 2012). These proteins fulfill important cellular functions in plants, often serving as the integrators of multiple regulatory and environmental signals (Sun *et al.*, 2013). They are abundant in the highly regulated processes, such as cellular signaling and transcription (Kragelund *et al.*, 2012). It has been estimated that 82–94% of all transcription factors, including those from *Arabidopsis thaliana*, contain extended IDRs (Liu *et al.*, 2006) and >70% of signaling proteins have long disordered regions (Iakoucheva *et al.*, 2002).

IDRs share some common sequence features such as low abundance of order-promoting and high abundance of disorder-promoting amino acids, low sequence complexity, poor sequence conservation, low mean hydrophobicity, high net charge, etc. (Dunker *et al.*, 2001; Nishikawa *et al.*, 2010; Romero *et al.*, 2001; Shimizu *et al.*, 2007; Uversky *et al.*, 2000). At the structural level, they are distinguished by the absence of stable secondary and/or tertiary structure, high flexibility, elevated β-sheet propensity and ability to undergo disorder-to-order transition when involved in protein–protein interactions (Dunker *et al.*, 2001; Uversky, 2011). These interactions are characterized by high specificity coupled with low affinity, ensuring their transient character, selectivity and reversibility. The specific sequence features together with the experimental structure-related databases have been used to develop specific predictors of intrinsic disorder. More than 50 tools for protein disorder prediction have been developed up to now (He *et al.*, 2009; Uversky, 2011).

Disordered protein regions are often involved in post-translational modifications (PTMs). It has been hypothesized, based on empirical evidence, that PTM sites are located predominantly in the easily accessible and flexible IDRs (Dunker *et al.*, 2002). Evidently, some advantages exist for positioning PTM sites within disordered regions. This location would facilitate the specific sequence-dependent association with modifying enzymes due to the high surface exposure and accessibility of the disordered regions. In addition, PTMs would typically cause only small changes on the surface of a structured protein, whereas large-scale structural changes, such as disorder-to-order transition, can be expected in a disordered region. The larger structural changes would generally elicit a deeper impact on protein function, ensuring a stronger regulatory effect of PTMs.

Numerous past studies addressed correlations between intrinsic disorder and individual PTMs in eukaryotic proteins. A well-established example includes protein phosphorylation. Several papers consistently reported that protein phosphorylation on Ser, Thr and Tyr occurs predominantly in the disordered regions of animal proteins (Fukuchi *et al.*, 2011; Gao *et al.*, 2010; Iakoucheva *et al.*, 2004; Yao *et al.*, 2012). Recently, this correlation has been confirmed in plant species using a predictive tool for analysis of protein phosphorylation in plants (Yao *et al.*, 2012). In addition, correlations between intrinsic disorder and glycosylation have also been investigated. Dissimilar structural preferences for the O-linked and N-linked protein glycosylation have been reported, reflecting differences in the catalytic mechanisms of these PTMs (Fukuchi *et al.*, 2011; Nishikawa *et al.*, 2010; Petrescu *et al.*, 2004). Also, several studies addressed correlations between intrinsic disorder and the universal Lys modifications, such as acetylation and methylation. It has been reported that acetylation and methylation showed a preference for occurrence in the disordered regions of animal proteins (Hansen *et al.*, 2006); however, these reports were challenged by other researchers (Pang *et al.*, 2007; Yao *et al.*, 2012).

In the present study, we analyzed proteome-wide correlations of protein disorder with the main types of eukaryotic PTMs, such as Ser/Thr/Tyr phosphorylation, Pro O-glycosylation, Asn N-glycosylation, Lys acetylation and Lys/Arg methylation in several typical monocotyledonous and dicotyledonous plant species. In agreement with the previous studies, we observed that phosphorylation, acetylation and O-glycosylation displayed a preference for occurrence in the disordered regions of plant proteins. However, in contrast to the previous results obtained using combined datasets of eukaryotic proteins, we found, based on the set of tools used in this study, that methylation tended to occur in ordered protein regions. Also, we found that N-glycosylation did not show a clear preference for either ordered or disordered regions of plant proteins.

## 2 METHODS

### 2.1 Datasets

In this study, the complete proteomes of dicotyledonous plant species, such as *A.thaliana* (thale cress) (Swarbreck *et al.*, 2008)*, Glycine max* (soybean) (Schmutz *et al.*, 2010)*, Populus trichocarpa* (poplar) (Tuskan *et al.*, 2006)*, Vitis vinifera* (grape) (Jaillon *et al.*, 2007), *Solanum lycopersicum* (tomato) (Sato *et al.*, 2012), as well as the proteomes of monocotyledonous plants, such as *Oryza sativa* (rice) (Ouyang *et al.*, 2007), *Brachypodium distachyon* (purple false brome) (Vogel *et al.*, 2010) and *Sorghum bicolor* (sorghum) (Paterson *et al.*, 2009), have been analyzed. The datasets covering the whole proteomes of the eight plant species were constructed using the proteome resources available at Phytozome (Goodstein *et al.*, 2012) (http://www.phytozome.net/). The redundancy check was executed using the OrthoMCL tool (Chen *et al.*, 2006) (http://orthomcl.org/cgi-bin/OrthoMclWeb.cgi?rm=orthomcl#Software) to remove the amino acid sequences with the pi_cutoff=90, pmatch_cutoff=90 and pv_cutoff=1e-30 options. The final non-redundant datasets of full proteome amino acid sequences were obtained by filtering out a small number of entries containing <50 and >2000 amino acids in length. The filtering statistics and the total numbers of sequences in the resulting datasets are presented in Supplementary Table S1. The average length of amino acid sequences in plant proteomes was calculated using Proteomix software (Chikayama *et al.*, 2004).

### 2.2 Prediction of protein disorder and PTMs

To calculate intrinsic disorder in plant proteins, the following predictive algorithms have been used in this study—RONN version 3 (Yang *et al.*, 2005), POODLE-L (Hirose *et al.*, 2007) and DISOPRED2 (Ward *et al.*, 2004b) provided online (http://www.strubi.ox.ac.uk/RONN, http://mbs.cbrc.jp/poodle/poodle.html and http://bioinf.cs.ucl.ac.uk/disopred/, respectively). The three predictive algorithms were reported to have comparable accuracy of 85–90%. RONN was used as the main predictor in this study because its scores were closer to the experimentally derived scores of intrinsic disorder for PDB-deposited protein structures. However, most of the results observed using RONN have also been confirmed with the two other tools and provided as Supplementary Data.

In this study, we analyzed four major types of eukaryotic protein PTMs, such as phosphorylation, glycosylation, methylation and acetylation. Specifically, Ser, Thr and Tyr phosphorylation, N-linked Asn glycosylation, O-linked Pro glycosylation, Lys/Arg methylation and Lys acetylation have been investigated. They have been predicted using the following bioinformatics tools freely available on the web.

Phosphorylation sites were predicted with the Musite tool (Gao *et al.*, 2010) downloaded from http://musite.sourceforge.net/. The sites of N-glycosylation were predicted with the NetNGlyc1.0 tool (R.Gupta *et al.* Unpublished data.) downloaded from http://www.cbs.dtu.dk/cgi-bin/nph-sw_request?netNglyc. The sites of acetylation were predicted using the online tool PAIL (Li *et al.*, 2006) (http://bdmpail.biocuckoo.org/), and the sites of methylation were identified with the web tool PMeS (Shi *et al.*, 2012) (http://bioinfo.ncu.edu.cn/inquiries_PMeS.aspx). The previously reported consensus sequence [A/S/T/V]-P(1,4)-X(0-10)-[A/S/T/V]-P(1,4) (Gomord *et al.*, 2010) was used to screen for the plant proteins containing sites of O-linked Pro glycosylation. The screening algorithm will be described in detail elsewhere.

### 2.3 PTM contents in ordered and disordered regions

Total numbers of specific PTM sites in ordered and disordered segments of the eight plant proteomes have been calculated using the employed predictive algorithms. These numbers were divided by the total numbers of amino acids in the ordered or disordered segment of each proteome, providing the values of normalized PTM contents. The relative abundance of a specific PTM in the disordered and ordered segments of plant proteomes was analyzed using the following ratio: Rd/o = Nd/Ld:No/Lo, where No is the total number of PTM sites in the ordered segment of a proteome, Lo is the length of the ordered proteome segment, Nd is the total number of PTM sites in the disordered segment of a proteome and Ld is the length of the disordered proteome segment. By this definition, the Rd/o value equals 1 if the relative abundances of a PTM in ordered and disordered regions are the same. It assumes a value of >1 when a PTM has a preference for occurrence in disordered regions, and it becomes <1 if a PTM tends to occur in ordered regions.

### 2.4 3D homology modeling of protein structure

Three-dimensional structure of IAA-alanine resistant gene 3 (IAR3) from *A.thaliana* (AT1G51760) was built by homology modeling based on the crystal structure of IAA-leucine resistant like gene 2 (ILL2) from *A.thaliana* (AT5G56660) resolved at a resolution of 2 Å. The coordinate file of the template was retrieved from the Protein Data Bank (PDB: 1xmb chain A). The modeled range covered residues 34–427, sequence identity between the model and its template was 58% (Supplementary Fig. S1). This sequence identity is considered to be high enough to make a reliable homology model. Homology modeling was carried out using the protein structure homology-modeling server SWISS-MODEL (Arnold *et al.*, 2006; Schwede *et al.*, 2003) (http://swissmodel.expasy.org/). Structure visualization and mapping of predicted PTM sites in this model was done with PYMOL (DeLano, 2002) (http://www.pymol.org/).

The generated model was validated using QMEAN analysis (Benkert *et al.*, 2008, 2009). The QMEAN score of the model, which ranges between 0 and 1, with higher values indicating better quality, was calculated to be 0.599, indicating good overall quality of the generated structure. The residues with the highest estimated errors were found to be located mainly in the loop regions of the computational model. The summary of the structural homology modeling is presented in Supplementary Figure S1.

### 2.5 Correlation analysis and statistical significance

The protein disorder degree has been correlated with the specific content of analyzed PTMs in plant proteins. The robustness of the observed correlations was confirmed by pairwise (disorder degree versus PTM content) regression analysis. The degree of correlation between the contents of various analyzed PTMs and protein disorder was evaluated by calculating Pearson correlation coefficients. The statistical significance of the Pearson correlation coefficients was determined by calculating one-tailed probability values, given the correlation value (*r*) and the sample size (*n*), with the significance level set to 0.05.

Calculations of correlation coefficients and *P*-values were performed using the online statistics calculators available at http://www.danielsoper.com/statcalc3/.

## 3 RESULTS

### 3.1 Comparison of datasets and global intrinsic disorder in plant proteomes

Plants are known to contain large gene families of closely related members due to frequent genome duplications. The number of sequences in the analyzed plant proteomes varied greatly from ∼26 000 entries in the grape *V.vinifera* to >51 000 sequences in the rice *O.sativa* (Supplementary Table S1). The redundancy check was carried out for every plant species studied to remove the amino acid sequences with >90% identity. As it could be expected, the redundancy of amino acid sequences was higher in large proteomes. Strong positive correlation has been observed between the content of redundant sequences and proteome size (Supplementary Fig. S2). We have further determined the average length of amino acid sequences in the plant proteomes, which varied from 345 amino acids in *O.sativa* to 421 amino acids in *B. distachyon* (Supplementary Table S1). In the following analysis, the number of predicted PTM sites in proteins was normalized to the uniform length of 400 amino acids, rather than per sequence, considering the difference in the average protein lengths in the datasets.

**Fig. 1.**
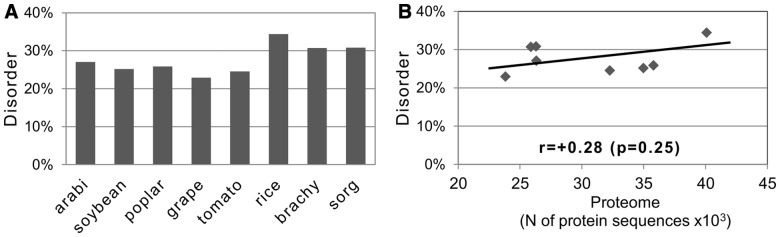
Evaluation of global protein disorder in plant proteomes. (**A**) Proteome disorder content was calculated with the RONN tool in the studied plant species. The results of pairwise correlation analysis between the disorder content and proteome size are shown in (**B**)

A significant variation in the degree of protein disorder has been observed using RONN among the proteomes of the analyzed plants, ranging from 23% in *V.vinifera* to ∼34% in *O.sativa* species (Fig. 1A). Notably, the disorder degree was significantly higher in monocots than in dicots (Supplementary Fig. S3A). Considering the importance of this finding, its statistical significance was independently confirmed using the alternative disorder prediction tools POODLE and DISOPRED (Supplementary Fig. S3B and C). The elevated disorder content in the rice *O.sativa*, as compared with other plant species, has also been reported by other studies (Fukuchi *et al.*, 2011; Xue *et al.*, 2012). In accordance with the previous report, that the content of intrinsic disorder is generally independent of the proteome size (Xue *et al.*, 2012), no significant correlation between the proteome size and disorder content has been observed in plants (Fig. 1B).

### 3.2 Correlation of intrinsic disorder with phosphorylation

Reversible protein phosphorylation is the most extensively studied PTM because it provides a major regulatory mechanism in eukaryotic cells. Phosphorylation was reported to be overrepresented in the disordered regions of eukaryotic proteins, including plants (Gao *et al.*, 2010; Iakoucheva *et al.*, 2004). Many sites of phosphorylation have been experimentally associated with the regions of intrinsic disorder. It has been reported that amino acid composition, sequence complexity, hydrophobicity, charge and other sequence attributes of regions adjacent to phosphorylation sites are similar to those of intrinsically disordered protein regions (Iakoucheva *et al.*, 2004), supporting the findings that phosphorylation occurs predominantly within IDRs.

**Fig. 2.**
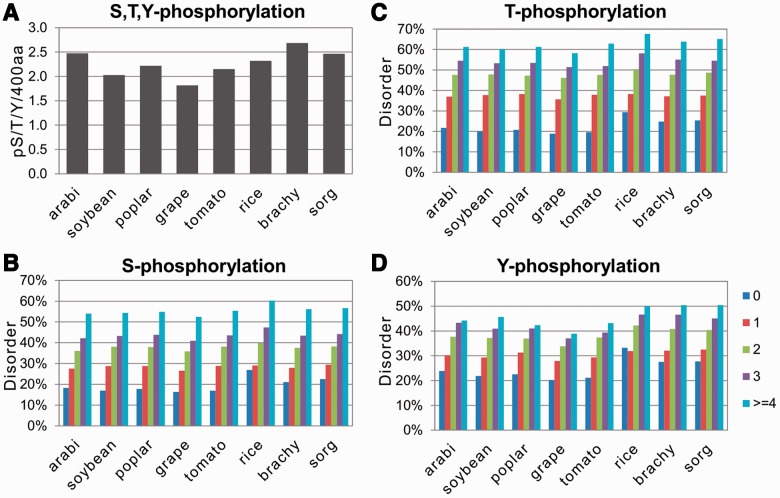
Correlations of protein disorder with the presence of residue-specific phosphorylation sites. Normalized content of phospho-S/T/Y sites in the studied plant proteomes is presented in (**A**). Relative rates of protein disorder in proteins with different numbers of predicted S, T and Y phosphorylation sites are presented in (**B**), (**C**) and (**D**), respectively

Previously, the bioinformatics tool Musite has been developed to predict phosphorylation sites based on the distinctive features of protein sequence (Gao *et al.*, 2010). It has been successfully applied to predict phosphorylation sites in plant proteins. It was reported that, for an overall plant model, Musite outperforms the plant-specific tools, such as PlantPhos (Lee *et al.*, 2011) and PhosPhAt (Durek *et al.*, 2010), in prediction accuracy (Yao *et al.*, 2012). An average accuracy of PlantPhos was estimated to be 82.4% for serine, 78.6% for threonine and 89.0% for tyrosine models (Lee *et al.*, 2011). In this work, we used the superior algorithm Musite to predict phosphorylation sites in the analyzed plant species. It was found that the average abundance of phosphorylation sites in different species varied on a small scale (Fig. 2A), suggesting common phosphorylation requirements in various plant species. Importantly, strong positive correlation was evident between protein disorder and phosphorylation. In every plant species analyzed, the positive correlation between the predicted number (from 0 to ≥4) of Ser, Thr or Tyr phosphorylation sites and the degree of intrinsic disorder has been observed (Fig. 2B–D). All of these correlations had a high statistical significance, as it could be judged from the one-tailed probability values of the calculated correlation coefficients (Table 1). Notably, the positive correlation between protein disorder and phosphorylation has also been confirmed when the alternative bioinformatics tool POODLE was used to predict the degree of intrinsic disorder (Supplementary Fig. S4). These data agree well with the previous reports. Thus, they provide a validation for the employed method of bioinformatics analysis.

**Table 1.** Statistical significance of correlations between protein disorder and predicted presence of PTMs

Species	pSer	pThr	pTyr	O-gly	N-gly	K-ace	R-met
*Arabidopsis*	**0.996**	**0.983**	**0.975**	**0.976**	0.692	**0.975**	**−0.893**
	**0.0002**	**0.0013**	**0.0024**	**0.0022**	0.0977	**<0.0001**	**0.0207**
Soybean	**0.993**	**0.970**	**0.989**	**0.981**	0.675	**0.959**	**−0.847**
	**0.0004**	**0.0031**	**0.0007**	**0.0016**	0.1056	**<0.0001**	**0.0351**
Poplar	**0.996**	**0.976**	**0.963**	**0.996**	**0.892**	**0.976**	**−0.923**
	**0.0002**	**0.0022**	**0.0042**	**0.0002**	**0.0209**	**<0.0001**	**0.0127**
Grape	**0.995**	**0.972**	**0.968**	**0.996**	0.789	**0.972**	**−0.914**
	**0.0002**	**0.0028**	**0.0034**	**0.0002**	0.0563	**<0.0001**	**0.0149**
Tomato	**0.993**	**0.975**	**0.969**	**0.991**	**0.875**	**0. 950**	**−0.854**
	**0.0004**	**0.0024**	**0.0033**	**0.0005**	**0.0260**	**0.0002**	**0.0327**
Rice	**0.979**	**0.998**	**0.951**	**0.922**	−0.450	**0.761**	−0.142
	**0.0018**	**<0.0001**	**0.0065**	**0.0129**	0.2235	**0.0140**	0.4099
*Brachypodium*	**0.992**	**0.995**	**0.992**	**0.952**	−0.040	**0.951**	−0.782
	**0.0004**	**0.0002**	**0.0004**	**0.0063**	0.4745	**0.0002**	0.0591
*Sorgham*	**0.993**	**0.994**	**0.996**	**0.957**	−0.301	**0.943**	−0.798
	**0.0004**	**0.0003**	**0.0002**	**0.0053**	0.3113	**0.0002**	0.0528

Pearson correlation coefficients and their statistical significance are presented above and below, respectively, for all analyzed correlations between protein disorder and PTMs. The column names stand for serine phosphorylation, threonine phosphorylation, tyrosine phosphorylation, O-linked glycosylation, N-linked glycosylation, lysine acetylation and lysine methylation in order.

### 3.3 Correlations of intrinsic disorder with glycosylation

Protein glycosylation is one of the most common PTMs in eukaryotic cells. The attachment of glycans affects protein folding, solubility and stability. It also alters the essential biological functions, such as immunogenicity, catalytic activity, clearance, ligand-receptor interactions, etc. (Webster and Thomas, 2012). Two major types of protein glycosylation have been discovered in eukaryotes, O-linked and N-linked glycosylation. The significant differences in O-glycosylation between plants and animals have been described, including the sites of glycan addition and glycan composition. It was found that in plants, glycans are mainly attached to hydroxylproline (Hyp). Protein disorder was suggested to facilitate hydroxylation of proline residues (Hon *et al.*, 2002). The addition of O-glycans to the hydroxyl group of Hyp is unique to plants. Although the target motif for Hyp-O-linked glycosylation in plants has been established (for details, see Sections 2 and 4), no bioinformatics tool is freely available as of now to predict O-glycosylation sites in plant proteins. In the present study, we used the previously reported consensus sequence to screen for the sites of Hyp-O-linked glycosylation.

**Fig. 3.**
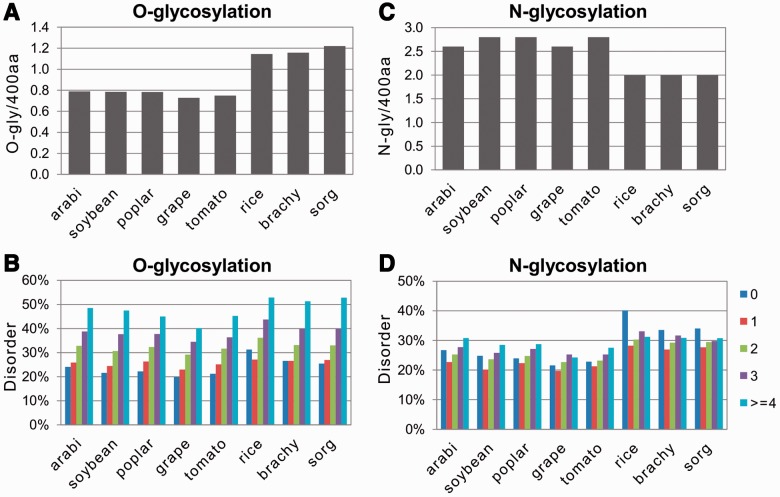
Correlations between protein disorder and glycosylation. Normalized contents of O-glycosylation and N-glycosylation in the studied plant proteomes are presented in (**A**) and (**C**), respectively. Relative rates of protein disorder in proteins with different numbers of predicted sites of O- and N-glycosylation are presented in (**B**) and (**D**), respectively

It was found that the average abundance of O-glycosylation varied significantly in the studied plant species from ∼0.8 to >1.2 sites per protein (Fig. 3A). The content of O-glycosylation was significantly higher in monocots than in dicots. Remarkably, the number of predicted sites for O-glycosylation correlated positively with the content of intrinsic disorder in plant proteins. In every plant species analyzed, positive correlation between the predicted number of O-glycosylation sites (from 0 to ≥4) and the content of intrinsic disorder has been observed (Fig. 3B). Similarly to phosphorylation, all of these correlations had a high statistical significance, as attested by the one-tailed probability values for the calculated correlation coefficients (Table 1). The positive correlation between protein disorder and O-glycosylation has also been confirmed with the alternative bioinformatics tool POODLE (Supplementary Fig. S5A).

Another major type of protein glycosylation in plants, N-linked glycosylation, is arguably the most conserved form of protein glycosylation in eukaryotes (Wilson, 2002). In plant cells, like in other eukaryotic cells, N-linked glycans are attached to the specific Asn residues in the consensus sequence of Asn-X-Ser/Thr, where X can be any amino acid except Pro. The N-linked glycosylation pathway in plants shares a high degree of homology with that in other eukaryotic organisms. Therefore, we used the general prediction algorithm NetNgly1.0 for predicting N-glycosylation in plant proteins. This tool revealed some degree of variation in the average abundance of N-glycosylation in the studied plant species (Fig. 3C). In general, the content of N-glycosylation was lower in monocots than in dicots. Importantly, unlike O-glycosylation, no universal tendency has been observed between the predicted presence of N-glycosylation sites and protein disorder. N-glycosylation tended to correlate positively with disorder content in the dicotyledonous species, such as *A.thaliana, G.max, P.trichocarpa, V.vinifera* and *S.lycopersicum*; however, the weak negative correlation between protein disorder and N-glycosylation was witnessed in the monocotyledonous *O.sativa*, *B.distachyon* and *S.bicolor* (Fig. 3D, Table 1). Notably, many of the observed correlations had a low statistical significance (Table 1). The correlations of N-linked glycosylation with protein disorder have also been confirmed using the alternative tool POODLE for predicting protein disorder (Supplementary Fig. S5B).

### 3.4 Correlations of intrinsic disorder with acetylation and methylation

Acetylation of internal lysines on ε-amino group and methylation on lysines and arginines are the highly reversible enzymatic reactions that change electrostatic properties of a protein molecule by neutralizing the positive charge of lysine and arginine residues. Alongside with protein phosphorylation, these PTMs play a major regulatory role in eukaryotic cells. The evidence has been presented that these PTMs may counteract phosphorylation, suggesting that the balance between phosphorylation and acetylation/methylation is important for physiologically relevant regulation (Kramer and Heinzel, 2010; Sakamaki *et al.*, 2011).

**Fig. 4.**
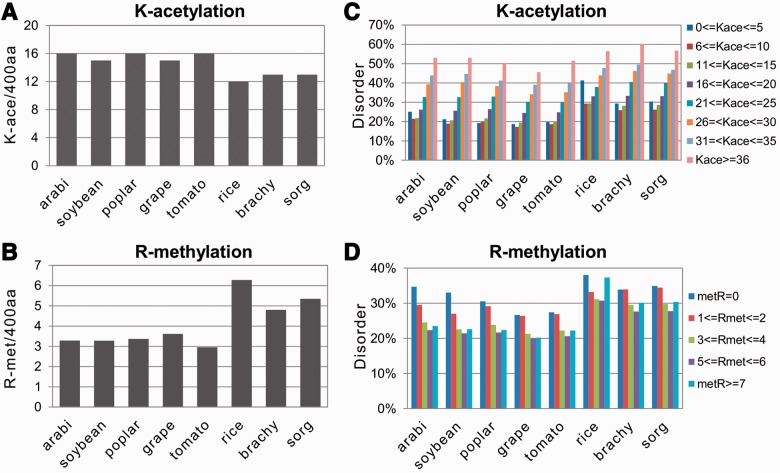
Correlations of protein disorder with acetylation and methylation. Normalized contents of K-acetylation and R-methylation in the studied plant proteomes are presented in (**A**) and (**C**), respectively. Relative rates of protein disorder in proteins with different numbers of predicted sites of K-acetylation and R-methylation are presented in (**B**) and (**D**), respectively

In the present work, we used the general acetylation prediction tool PAIL, freely available on the Internet, to predict the sites of lysine acetylation. The accuracies of PAIL were reported to be 85.13, 87.97 and 89.21% at low, medium and high thresholds, respectively (Li *et al.*, 2006). This algorithm was trained on a set of experimentally verified acetylation sites from different eukaryotic proteins belonging to various biological species, including plants. We have found that the content of acetyllysine varied in the analyzed plant species from 12 to 16 sites per protein (Fig. 4A). The content of lysine acetylation was markedly lower in monocots than in dicots. A significant correlation has been observed between the predicted presence of lysine acetylation sites and protein disorder. Contents of acetyllysine and disorder correlated positively in all plant species analyzed (Fig. 4B). Importantly, all of these correlations were statistically significant at 95% confidence level (Table 1).

Methylation of internal lysines and arginines is a PTM commonly found in proteins associated with nucleic acids, such as histones and transcriptional regulators. We have found that this modification displayed a negative correlation with protein disorder in the analyzed plant species, suggesting that it has a preference for occurrence in ordered regions (Fig. 4D and Supplementary Figures S6B and S7B). In every plant species analyzed, the negative correlation between the predicted number of methylation sites (from 0 to ≥7) and protein disorder has been observed. Most of these correlations were statistically significant, as confirmed by the one-tailed probability values of the correlation coefficients (Table 1). Notably, in contrast to lysine acetylation and lysine methylation, the content of arginine methylation was found to be significantly higher in monocots than in dicots (Fig. 4C). Most probably, this can be attributed to the relative abundance of lysine and arginine in the plant species analyzed. We have found that lysine content is decreased and arginine content is increased in the monocotyledonous plants, as compared with those in the dicotyledonous plants (data not shown). The observed correlations of acetylation and methylation with protein disorder have also been confirmed with the alternative bioinformatics tool POODLE (Supplementary Fig. S7).

### 3.5 Relative contents of PTMs in ordered and disordered regions of plant proteins

To independently verify the observed correlations, relative abundances of specific PTMs in ordered and disordered regions of plant proteins have been determined. The values of the relative abundances were presented as a ratio of normalized PTM contents (Rd/o) in disordered and ordered segments of plant proteomes (for details, see Section 2.3). The results of these calculations are shown in Table 2. In general, they confirm the major findings of correlation analysis, such as the preference of phosphorylation, O-glycosylation and acetylation for occurrence in disordered regions and the opposite tendency for methylation. Moreover, this approach allowed us to estimate the robustness of the observed correlations. For instance, high values of the Rd/o parameter obtained for phosphorylation and O-glycosylation (≥2.5 and ≥5.2, respectively) were indicative of robust relationships, whereas the correlation between protein disorder and acetylation was less robust, as it could be judged from the lower Rd/o value (≥1.6) calculated for this PTM (Table 2). Similarly, arginine methylation, which has a preference for occurrence in ordered protein regions, displayed more robust correlation in dicots than in monocots, as it is suggested by Rd/o values (Table 2). This finding is consistent with the results of correlation analysis, indicating a higher statistical significance of this correlation in dicots (Table 1). Notably, in contrast to correlation analysis that failed to detect any universal preference for N-glycosylation (Table 1), the alternative approach revealed that this PTM favors disordered regions in all plant species analyzed (Table 2). Possible explanation for this discrepancy is provided further in Section 4 based on statistical significance of the obtained results.

**Table 2.** Relative PTM contents in ordered and disordered segments of plant proteomes

Species category	S,T,Y-phospho	O-gly	N-gly	K-ace	R-met	K-met
Dicots	≥3.0	≥5.2	≥1.6	≥1.8	≤0.50	≤0.64
Monocots	≥2.5	≥6.2	≥1.2	≥1.6	≤0.88	≤0.68

The ratios of normalized PTM contents (Rd/o), calculated as described in the Section 2.3, are presented in the table.

### 3.6 Mapping PTMs on a protein structure

**Fig. 5.**
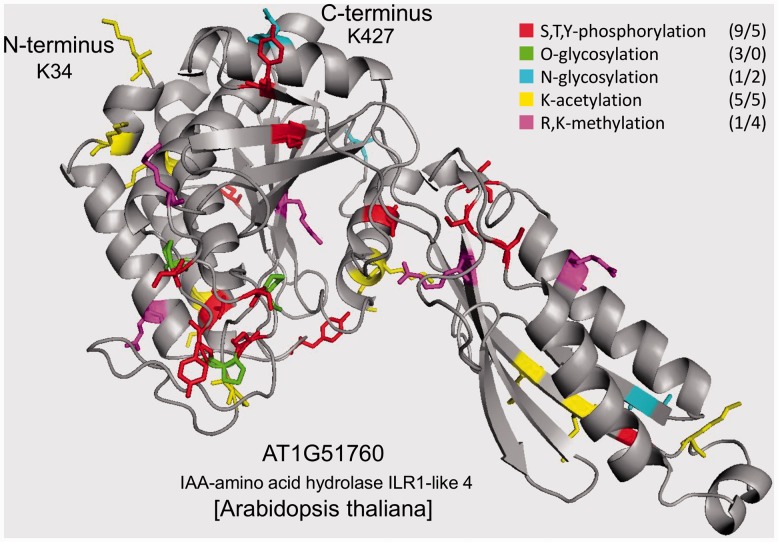
Modeled 3D structure of the plant protein IAA-amino acid hydrolase from *A.thaliana* with mapped sites of analyzed PTMs. The statistics in the right upper corner shows the disorder-to-order ratio of PTM occurrence in the protein molecule

To highlight the correlations observed, the predicted sites of PTMs analyzed in this study were mapped onto the modeled 3D structure of the plant protein IAA-amino acid hydrolase from *A.thaliana* (Fig. 5). The PTM sites in this protein have been predicted with the bioinformatics tools used in the present study. The protein structure was built by homology modeling based on the crystal structure of IAA-leucine resistant like gene 2 (ILL2) (AT5G56660). The summary and validation of the molecular modeling are presented in Supplementary Figure S1 (see Section 2 for more details). All in all, 35 putative PTM sites have been mapped in this protein. The statistics of their occurrence in the ordered and disordered regions of the protein molecule is presented in the right up corner of Figure 5. It agrees well with the correlations revealed by this study. Most notably, the sites of phosphorylation and O-glycosylation are mapped preferentially in unstructured regions, the sites of N-glycosylation and acetylation display no clear preference for either ordered or disordered sequence, and the sites of methylation are mapped mainly in the structured regions of the protein molecule. The model illustrates well the major findings of this study.

## 4 DISCUSSION

In this work, we examined the correlations between protein disorder and multiple PTMs in eight monocotyledonous and dicotyledonous plant species. Previous studies suggested that enzyme-mediated reversible PTMs have a preference for surface accessible and disordered environments. In accordance with this idea, we have found that disorder content correlates positively with the presence of predicted phosphorylation sites in plant proteins (Fig. 2 and Supplementary Fig. S4; Table 2). The positive correlations between protein disorder and Ser, Thr and Tyr phosphorylation had a high statistical significance (Table 1). These observations are in good agreement with the previous studies of eukaryotic proteins from animal species. For instance, data mining of human proteome revealed that phosphorylation occurs two to three times more often within disordered than ordered regions (Fukuchi *et al.*, 2011). The same magnitude of difference has been observed in our present study using calculation of relative PTM abundance in ordered and disordered segments of plant proteomes (Table 2). In addition, similar results were obtained previously on the investigation of PDB-annotated structures of plant proteins with the disorder-assisted tool for prediction of phosphorylation sites Musite (Yao *et al.*, 2012). Thus, it can be concluded that the analytic approach applied in our study is adequate in general, considering high consistency of the obtained results on phosphorylation.

It has been reported previously that the sites of O-linked glycosylation are predominantly located in the IDRs of mammalian proteins (Fukuchi *et al.*, 2011; Nishikawa *et al.*, 2010). In the present study too, the strong positive correlation between protein disorder and O-glycosylation has been evidenced in plant proteins (Fig. 3B and Supplementary Fig. S5A; Tables 1 and 2). The existence of this correlation should be attributed, in the first place, to the distinctive pattern of O-glycosylation in plants, which occurs predominantly on Pro residues. The target motif of O-glycosylation has been found to include at least two [A/S/T/V]-P repeats (Tan *et al.*, 2003). The presence of multiple Pro residues in the regions of plant O-glycosylation consensus sequence [A/S/T/V]-P(1,4)-X(0-10)-[A/S/T/V]-P(1,4) (Gomord *et al.*, 2010) makes the formation of secondary structure in these regions virtually impossible. Hence, the positive correlation between protein disorder and O-glycosylation in plants could be expected.

On the other hand, N-glycosylation is known to occur co-translationally before a protein is fully folded. This should result in the lack of any structural preference for this modification. In accordance with this consideration, no clear structural preference has been reported for N-glycosylation in animal proteins (Petrescu *et al.*, 2004). Consistently, the correlation analysis performed in our study also failed to reveal a strong universal unidirectional relationship between N-glycosylation and protein disorder in plant proteins (Fig. 3D, Supplementary Fig. S5B). Instead, N-glycosylation was found to correlate with disorder content positively in dicotyledonous plant species and negatively in monocotyledonous species, both with a low statistical significance, suggesting species-specific patterns of N-glycosylation in plants (Table 1). It should be noted in this connection that previous studies pointed to the existence of species-, organ- and development-specific N-glycosylation patterns in plants (Gomord *et al.*, 2010). Thus, it can be hypothesized that the modifying enzyme, oligosaccharyltransferase, may display selective recognition for the specific modification sites in different plant species. Still, at present, no experimental evidence for existence of these differences has been presented and there is obviously a need for systematic studies on the glycosylation patterns in various plant species.

Notably, in contrast to correlation analysis, the alternative approach based on the calculation of relative PTM abundance in ordered and disordered segments of plant proteomes revealed that this PTM has a weak preference for disordered sequence in all plant species analyzed (Table 2). Presumably, this discrepancy may be attributed to the low accuracy of the NetNGlyc predictor. Its reported overall accuracy was the lowest among all the tools used, reaching only 76% in a cross-validated experiment (http://www.cbs.dtu.dk/services/NetNGlyc/abstract.php). Considering that the reported predictive accuracy of RONN is ∼85% (Yang *et al.*, 2005), it can be approximated that the Rd/o values within 0.6–1.4 may not be statistically significant. Thus, the correlations between N-glycosylation and disorder observed in monocots should be treated as unreliable, whereas the correlations in dicots are of low robustness, as it is suggested by the Rd/o values (Table 2). Consistently, the similar conclusion can be drawn based on the statistical significance of Pearson coefficients obtained by correlation analysis (Table 1).

It has been reported previously that acetyllysine is more likely to be found within surface-accessible and disordered protein regions, whereas methyllysine does not show any significant preference for surface accessibility or intrinsic disorder (Pang *et al.*, 2007). On the contrary, it was found on building an SVM predictor for protein methylation, that both Arg and Lys methylation sites are likely to be intrinsically disordered (Daily *et al.*, 2005). The most recent study of correlations between PTMs and intrinsic disorder reported that methylation had a preference for occurrence in disordered regions and acetylation did not show any significant preference for either disordered or ordered regions (Gao and Xu, 2012). Now, our present work demonstrates that methylation tends to occur in ordered regions, whereas acetylation shows a preference for disordered regions (Fig. 4, Supplementary Figs S6 and S7; Tables 1 and 2). The reason for discrepancy of the results obtained in these studies is presently unclear.

Most probably, this inconsistence can be attributed to the drastic difference in the analyzed datasets. In the previous studies, the datasets included proteins from various eukaryotic species, whereas our study was conducted on the individual plant proteomes. Notably, combining the entries from different species in one dataset may be detrimental to correlation analysis. Opposite correlations between the contents of protein disorder and specific PTM can be observed among the individual plant species. For instance, the negative correlation between protein disorder and N-glycosylation has been revealed in the monocotyledonous *O.sativa*, *B.distachyon* and *S.bicolor*; however, the opposite tendency was evident in the dicotyledonous *A.thaliana, G. max, P.trichocarpa, V.vinifera* and *S.lycopersicum* (Fig. 3D, Supplementary Fig. S5B; Table 1). At present, no other comparable data are available to elaborate this issue, as the correlations between PTMs and protein disorder in the individual plant species have not been investigated and compared before. Additional studies are required to confirm the observed tendencies in the particular plant species using various alternative bioinformatics and experimental approaches.

Another factor that can potentially compromise the performed correlation analysis is applicability of the used prediction algorithms for analysis of plant proteins. In this connection, it should be noted that both the methylation predictor PMeS and the acetylation predictor PAIL were trained on the databases that included plant proteins. The tools were annotated as the general PTM predictors for eukaryotic proteins. Admittedly, these algorithms have not been comprehensively applied to plants and their performance exclusively for plant proteins have not been evaluated. Still, it is hard to acclaim that both tools systematically misrepresent the modification sites in certain plant proteins, as this problem has never come to light before in the related studies. In addition, it is unlikely that observed discrepancy with the previously obtained results is introduced by a bias of the protein disorder prediction algorithm RONN, as the observed correlations have been confirmed with the alternative disorder prediction tool POODLE.

Thus, the surprising finding of our study that methylation has a preference for occurrence in the ordered protein regions, reflects most probably a *bona fide* tendency in the investigated plant species. This finding was confirmed independently by both correlation analysis (Table 1) and by calculation of methylation content in the ordered and disordered segments of plant proteomes (Table 2). Moreover, the observed tendency concerned both Arg and Lys methylation sites (Fig. 4, Supplementary Fig. S7; Table 2). In this connection, the PMeS prediction algorithm achieved the accuracies of 92.82 and 89.16% for arginine and lysine, respectively (Shi *et al.*, 2012). Notably, Arg methylation was found to be more abundant than Lys methylation (Fig. 4B, Supplementary Fig. S6A); the two PTMs are known to be catalyzed by different enzymes. Previously, the studies of PRMT1, the major methyltransferase responsible for ∼85% of total Arg methylation in mammalian cells, demonstrated that this enzyme has a broad substrate specificity, with a preference for Arg residues flanked by one or more Gly residues (Gary and Clarke, 1998). More recent studies found that some distal substrate residues also affect Arg methylation (Osborne *et al.*, 2007), suggesting the existence of additional recognition determinants for this enzyme. Notably, PRMT1 homologs have been biochemically characterized in *A.thaliana* and *O.sativa* (Ahmad *et al.*, 2011; Yan *et al.*, 2007). In light of our findings, it is tempting to surmise that both Arg- and Lys-specific plant methylases may recognize some structural determinants of modification sites, directing their specificity toward the substrate residues located in the ordered regions of plant proteins. Presently, no experimental evidence has been presented for existence of the additional structural recognition determinants of methylation in plants.

In conclusion, our study demonstrates that some PTMs, such as phosphorylation, O-glycosylation and acetylation, display a clear preference for occurrence in disordered regions of plant proteins. However, the opposite tendency is evident for methylation, and N-glycosylation does not display a universal preference for either ordered or disordered protein regions. Also, our analysis reveals the marked differences between the integral characteristics of monocot and dicot proteomes. They include elevated disorder degree, increased rate of O-glycosylation and R-methylation, decreased rate of N-glycosylation, K-acetylation and K-methylation in monocotyledonous plant species, as compared with dicotyledonous species. To our knowledge, this is the first time when these differences are brought to light in a genome-wide study of plant proteins. The differences observed should reflect the large variation in the major PTM systems of monocotyledonous and dicotyledonous plants. At present, the evolutionary and environmental background underlying this variation is not established.

## Supplementary Material

Supplementary Data
