# Supplementary material for: Correlations between predicted protein disorder and post-translational modifications in plants
Source: Bioinformatics. 2014 Jan 7;30(8):1095–103. doi: 10.1093/bioinformatics/btt762 (PMC3982157; doi:10.1093/bioinformatics/btt762)
Supplement: Supplementary Data [file supp_30_8_1095__index.html]

Correlations between predicted protein disorder and post-translational modifications in plants — Correlations between predicted protein disorder and post-translational modifications in plants — Supplementary Data 

# Correlations between predicted protein disorder and post-translational modifications in plants

## Supplementary Data

files

**Files in this Data Supplement:**

- Supplementary Data - tif file
- Supplementary Data - tif file
- Supplementary Data - tif file
- Supplementary Data - tif file
- Supplementary Data - tif file
- Supplementary Data - tif file
- Supplementary Data - tif file
- Supplementary Data - doc file
