# Supplementary figures and images for: Correlations between predicted protein disorder and post-translational modifications in plants
Source: Bioinformatics. 2014 Jan 7;30(8):1095–103. doi: 10.1093/bioinformatics/btt762 (PMC3982157; doi:10.1093/bioinformatics/btt762)

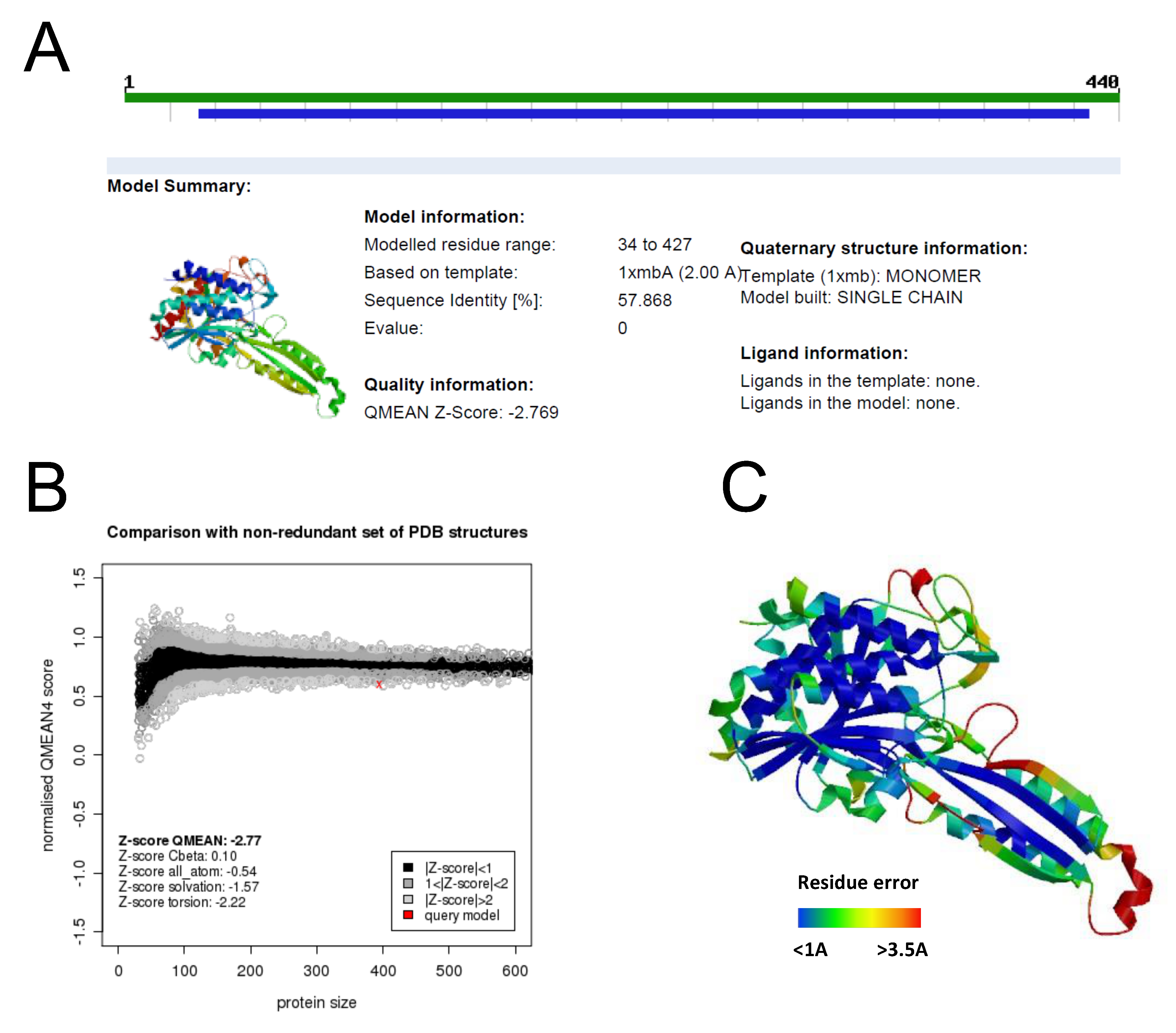

Supplement: Supplementary Data [file supp_btt762_Fig_S1.tif]

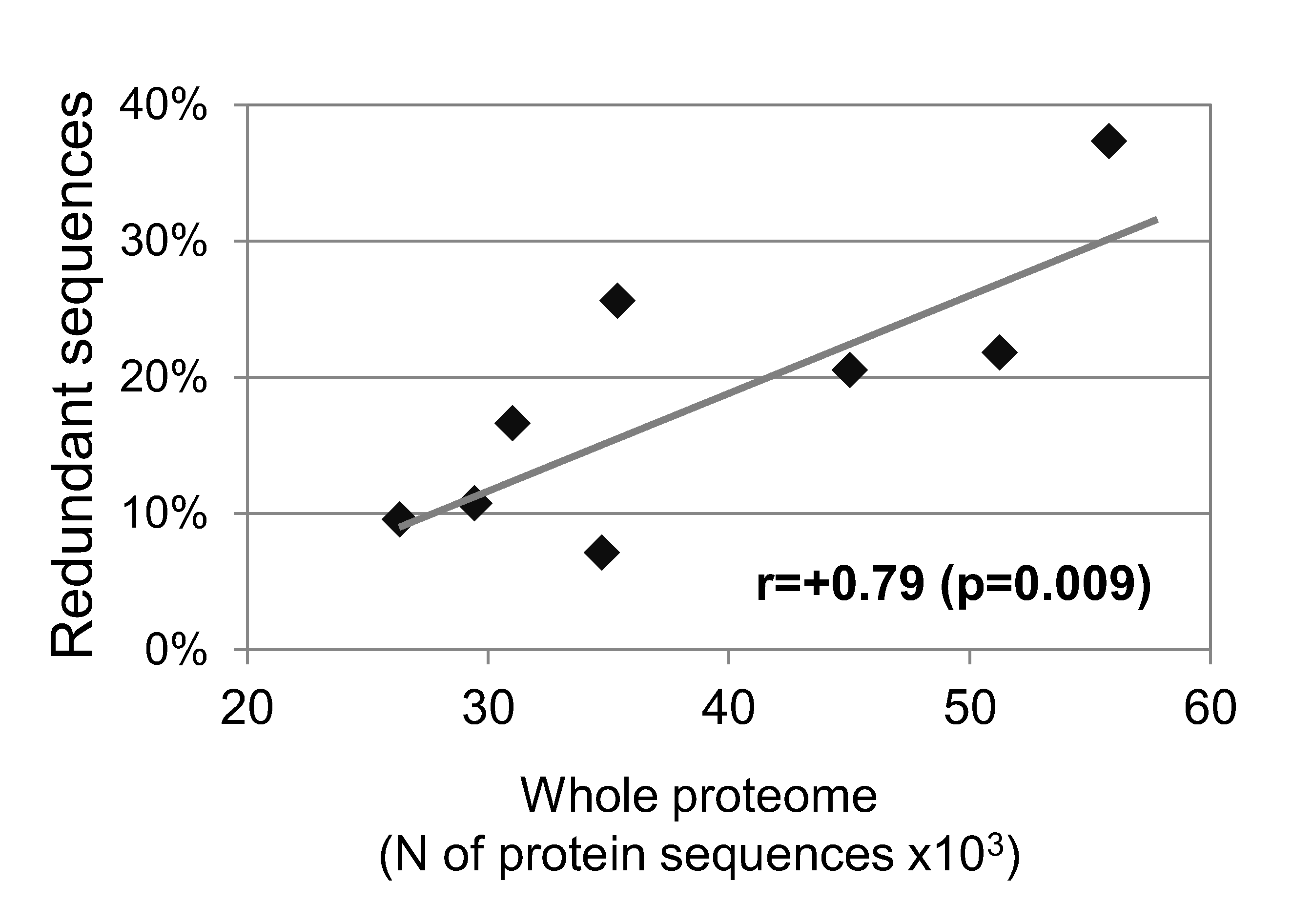

Supplement: Supplementary Data [file supp_btt762_Fig_S2.tif]

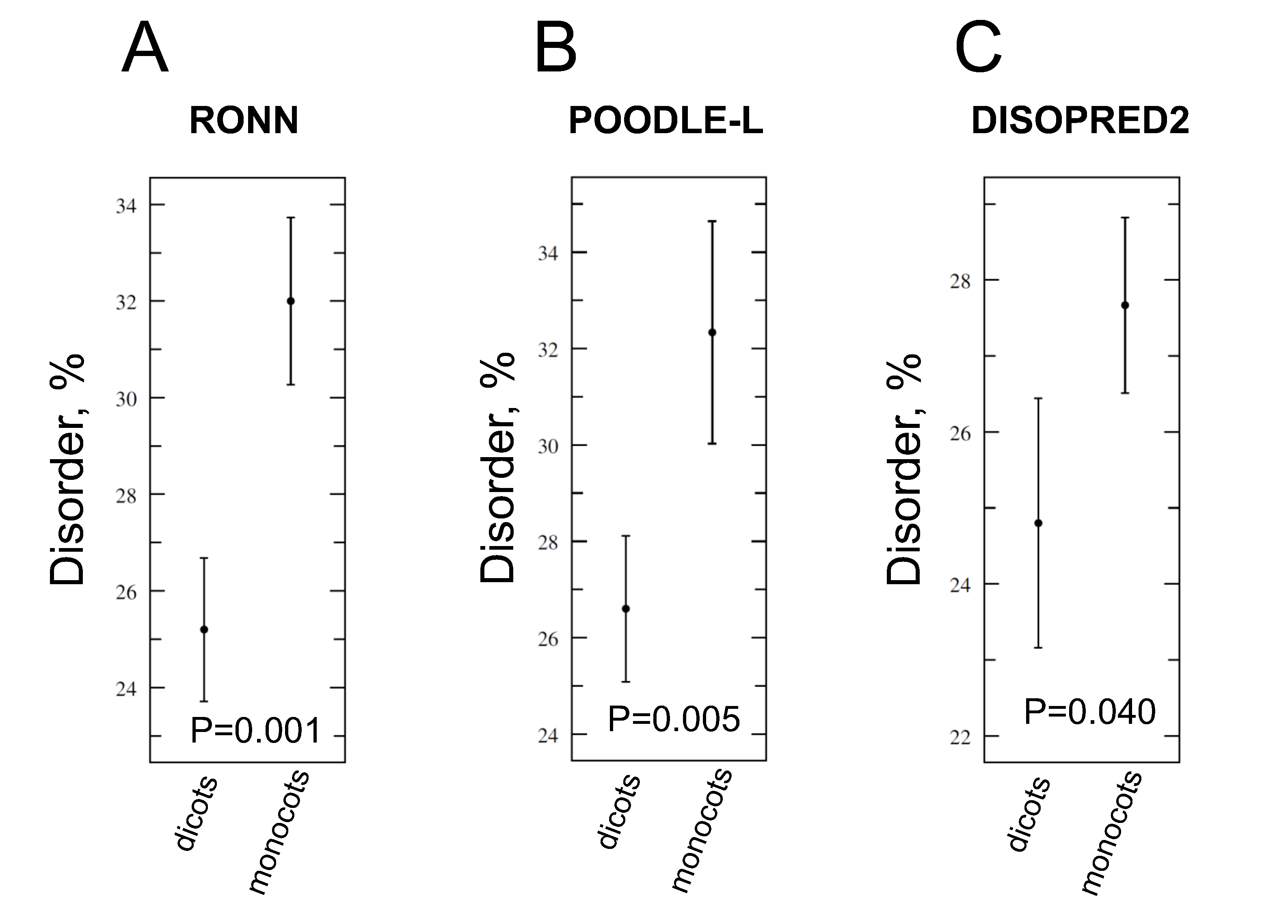

Supplement: Supplementary Data [file supp_btt762_Fig_S3.tif]

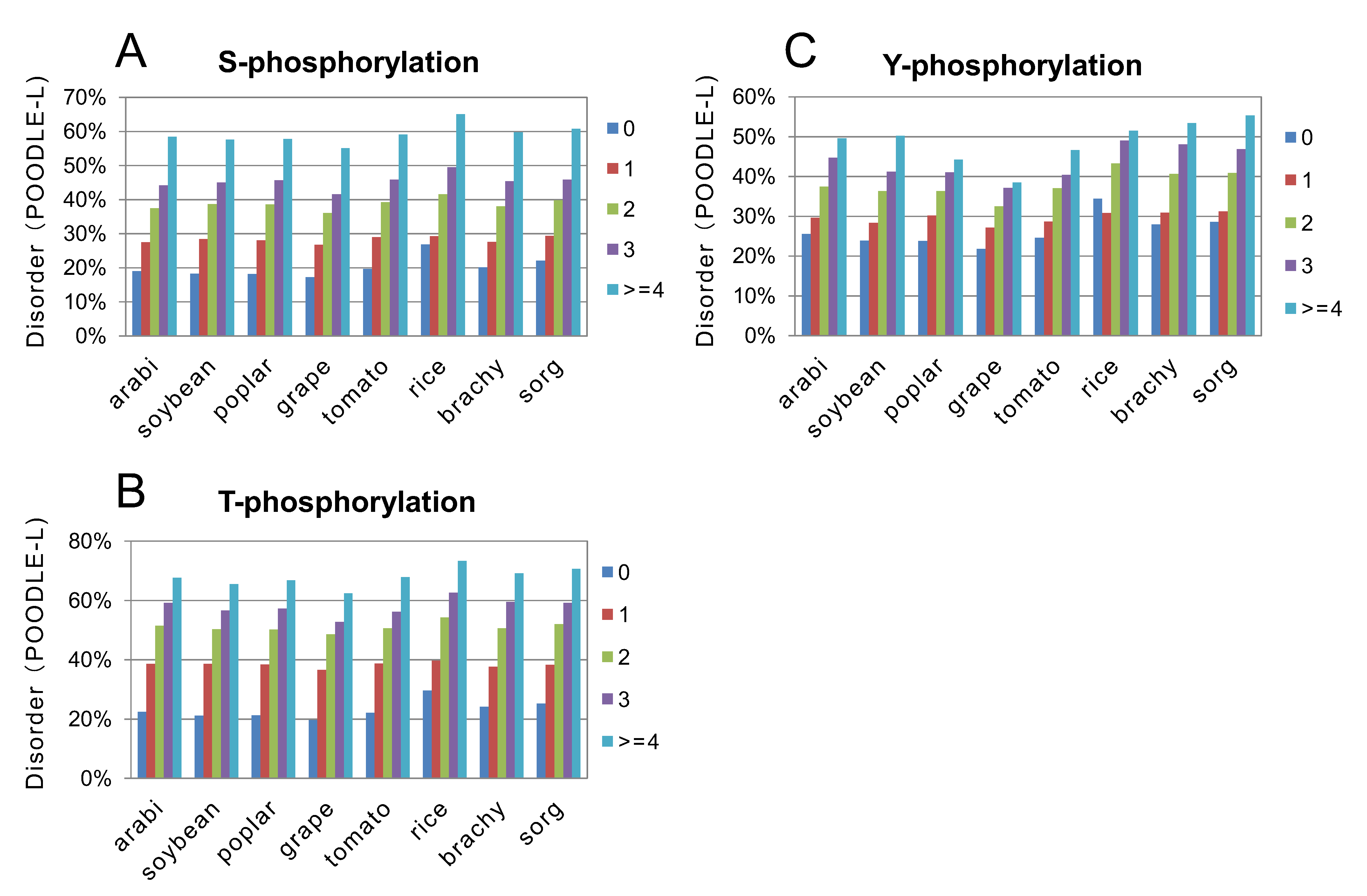

Supplement: Supplementary Data [file supp_btt762_Fig_S4.tif]

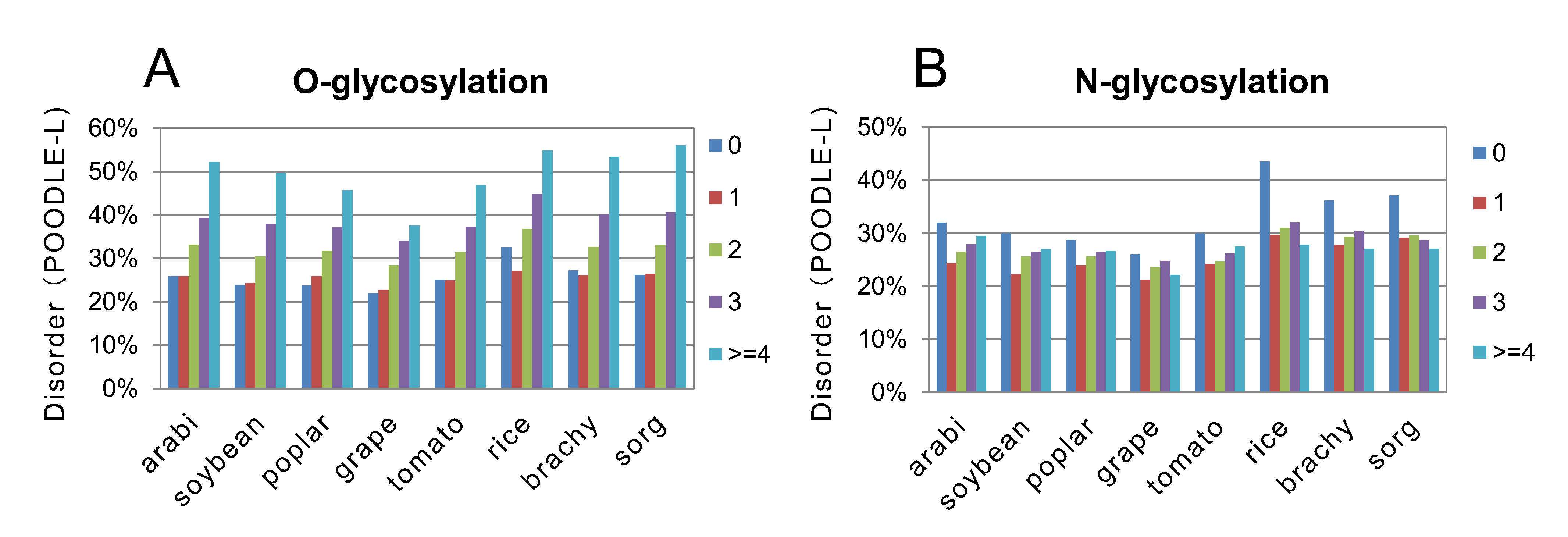

Supplement: Supplementary Data [file supp_btt762_Fig_S5.tif]

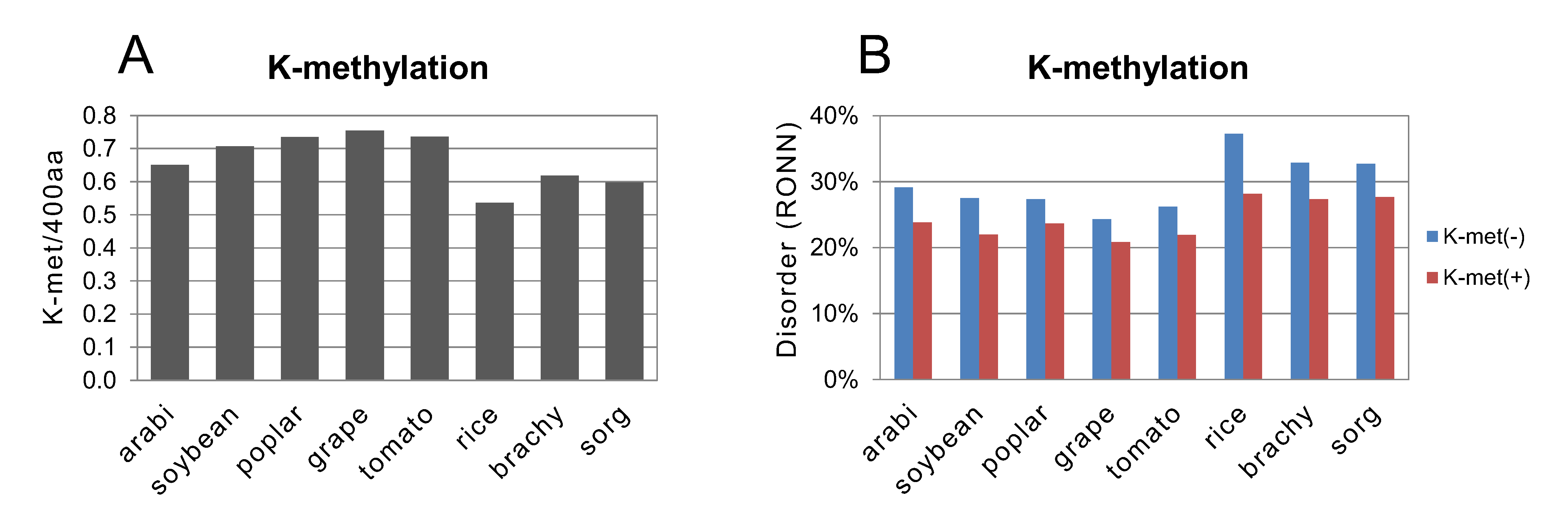

Supplement: Supplementary Data [file supp_btt762_Fig_S6.tif]

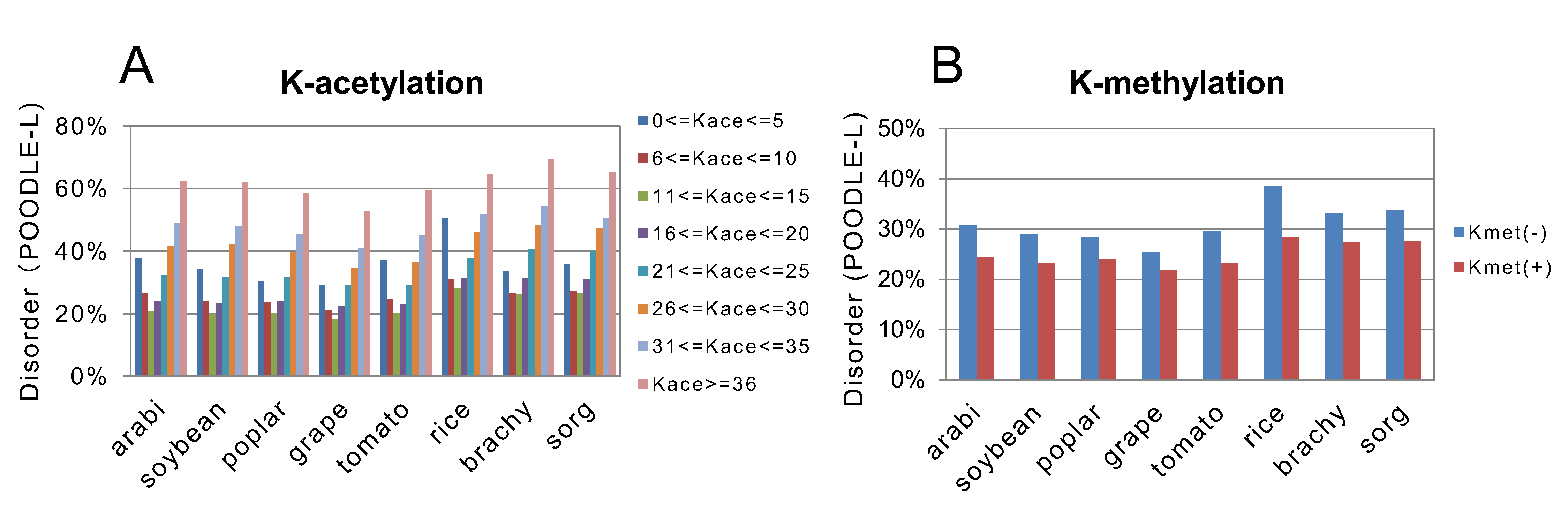

Supplement: Supplementary Data [file supp_btt762_Fig_S7.tif]
