# Supplementary material for: Correlations between predicted protein disorder and post-translational modifications in plants
Source: Bioinformatics. 2014 Jan 7;30(8):1095–103. doi: 10.1093/bioinformatics/btt762 (PMC3982157; doi:10.1093/bioinformatics/btt762)
Supplement: Supplementary Data [file supp_btt762_Supplementary_Table_Legends.doc]

**Supplementary Figure/Table legends**

**Table S1.** Total numbers and average lengths of protein sequences in the analyzed plant proteomes. Data filtering was performed as described in “METHODS”.

**Fig. S1.** Structural homology modeling of the plant protein IAA-amino acid hydrolase from *Arabidopsis thaliana.* Model summary is presented in **(A)** and estimation of the QMEAN score for the generated 3D model is shown in **(B)**. Estimated residue error in the model is visualized using a color gradient in panel **(C)**.

**Fig. S2.** Correlation of protein redundancy with proteome size. The result of pair-wise correlation analysis between the proteome size and protein redundancy in the studied plant species is presented. Correlation coefficient and its one-tailed probability values are indicated.

**Fig. S3.** Statistical significance of the difference between disorder degrees of monocot and dicot proteins. Disorder degree in the analyzed plant proteomes was calculated using the predictive tools RONN **(A)**, POODLE-L **(B)** and DISOPRED2 **(C)**.

**Fig. S4.** Correlations of protein disorder with the presence of residue–specific phosphorylation sites. Relative rates of protein disorder, calculated with the POODLE-L tool in proteins with different numbers of predicted S, T and Y phosphorylation sites, are presented in panels **(A)**, **(B)**, and **(C)**, respectively.

**Fig. S5.** Correlations of protein disorder with glycosylation. Relative rates of protein disorder, calculated with the POODLE-L tool in proteins with different numbers of predicted sites of O- and N-glycosylation, are presented in **(A)** and **(B)**, respectively.

**Fig. S6.** Correlations of protein disorder with K-methylation. Normalized content of K-methylation sites in the studied plant proteomes is presented in **(A)**. Relative rates of disorder in the proteins with or without the predicted sites of K-methylation are shown in **(B)**. Disorder degree was predicted using RONN.

**Fig. S7.** Correlations of protein disorder with acetylation and methylation. Relative rates of disorder in proteins with different numbers of predicted sites of K-acetylation and K-methylation are presented in **(A)** and **(B)**, respectively. Disorder degree was predicted using POODLE-L.

**Table S1.**

| **Arabi** | **Soybean** | **Poplar** | **Grape** | **Tomato** | **Rice** | **Brachy** | **Sorgham** |
| --- | --- | --- | --- | --- | --- | --- | --- |
| **Number of sequences** | | | | | | | |
| **before filtering** | | | | | | | |
| 35,386 | 55,787 | 45,033 | 26,346 | 34,727 | 51,258 | 31,029 | 29,448 |
| **after filtering** | | | | | | | |
| 26,326 | 34,972 | 35,791 | 23,829 | 32,257 | 40,087 | 25,875 | 26,293 |
| **Average length, aa** | | | | | | | |
| 402 | 398 | 369 | 373 | 351 | 345 | 421 | 416 |
